# CleanBar: a versatile demultiplexing tool for split-and-pool barcoding in single-cell omics

**DOI:** 10.1093/ismeco/ycaf134

**Published:** 2025-08-01

**Authors:** Vicente Arnau, Alicia Ortiz-Maiques, Juan Valero-Tebar, Lucas Mora-Quilis, Vaida Kurmauskaite, Lorea Campos Dopazo, Pilar Domingo-Calap, Mária Džunková

**Affiliations:** Institute for Integrative Systems Biology (I2SysBio), University of Valencia and Spanish National Research Council (CSIC), 46980, Paterna, Valencia, Spain; Institute for Integrative Systems Biology (I2SysBio), University of Valencia and Spanish National Research Council (CSIC), 46980, Paterna, Valencia, Spain; Institute for Integrative Systems Biology (I2SysBio), University of Valencia and Spanish National Research Council (CSIC), 46980, Paterna, Valencia, Spain; Institute for Integrative Systems Biology (I2SysBio), University of Valencia and Spanish National Research Council (CSIC), 46980, Paterna, Valencia, Spain; Atrandi Biosciences, Savanorių pr. 178C-101, LT-03154 Vilnius, Lithuania; Atrandi Biosciences, Savanorių pr. 178C-101, LT-03154 Vilnius, Lithuania; Institute for Integrative Systems Biology (I2SysBio), University of Valencia and Spanish National Research Council (CSIC), 46980, Paterna, Valencia, Spain; Institute for Integrative Systems Biology (I2SysBio), University of Valencia and Spanish National Research Council (CSIC), 46980, Paterna, Valencia, Spain

**Keywords:** split-and-pool barcoding, microbial single-cell genomics, phage-bacteria interactions, demultiplexing, Atrandi, PacBio

## Abstract

Split-and-pool barcoding generates thousands of unique barcode strings through sequential ligations in 96-well plates, making single-cell omics more accessible, thus advancing microbial ecology, particularly in studies of bacterial interactions with plasmids and bacteriophages. While the wet-lab aspects of the split-and-pool barcoding are well-documented, no universally applicable bioinformatic tool exists for demultiplexing single cells barcoded with this approach. We present CleanBar (https://github.com/tbcgit/cleanbar), a flexible tool for demultiplexing reads tagged with sequentially ligated barcodes, accommodating variations in barcode positions and linker lengths while preventing misclassification of natural barcode-like sequences and handling diverse ligation errors. It also provides statistics useful for optimizing laboratory procedures. We demonstrate CleanBar’s performance with the Atrandi platform for microbial single-cell genomics, coupled with PacBio sequencing, to reach a cell throughput comparable with traditional bulk metagenomics, but overcoming its limitations in studying phage-bacteria interactions. In four *Klebsiella* strains infected with their corresponding phages and a control phage, the single-cell genomics revealed infection heterogeneity and enabled phage copy number estimation per cell. By combining efficiency, adaptability, and precision, CleanBar, when applied to the Atrandi split-and-pool barcoding platform and PacBio sequencing, serves as a powerful high-throughput tool for advancing microbial single-cell genomics and understanding microbial ecology and evolution.

## Graphical Abstract

**Graphical Abstract:**
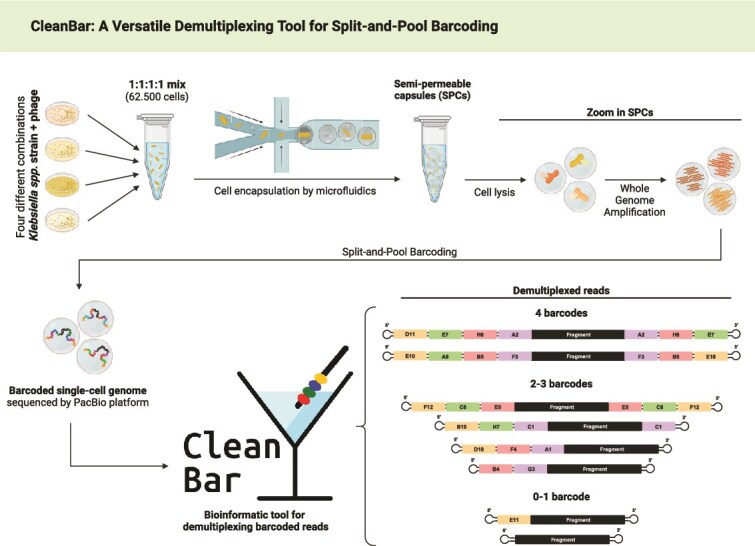

## Introduction

Single-cell omics have transformed our ability to resolve cellular genetic and functional heterogeneity, providing insights into complex biological systems that are often masked in bulk analyses. Complete reference genomes of plants and animals [1, 2] and straightforward eukaryotic reverse transcription chemistry [3] have advanced the development of high-throughput single-cell microfluidic platforms, such as 10X Genomics and SeekGene [4–6], followed by the emergence of platforms for prokaryotes, such as M20 Genomics [7]. These platforms involve direct barcoding of thousands of single-cells during the processing of droplets with specialized microfluidic equipment, which limits the flexibility of their protocols for processing unconventional sample types. As a result, prokaryotic research, which often deals with microbiomes composed mainly of previously unknown taxa, still largely relies on data obtained from bulk omics. Although advancements in meta-omics have facilitated the identification of new microbial taxa [8], bulk approaches are inefficient in associating bacteria with their plasmids and phages [9], which often carry auxiliary metabolic genes essential for microbial survival, highlighting unexplored avenues in microbial ecology and evolution [10, 11]. Single-cell genomics, however, enables the detection of these mobile genetic elements within individual microbial cells [12, 13]. Yet, its broader application of microbial single-cell genomics has been limited by challenges in handling small reagent volumes applied to individual cells sorted into 96- or 384-well plates [14], as well as the high costs of processing each cell as a separate sequencing library, with <30% of amplified cells meeting high genome quality standards for completeness and contamination [15–17].

**Figure 1:**
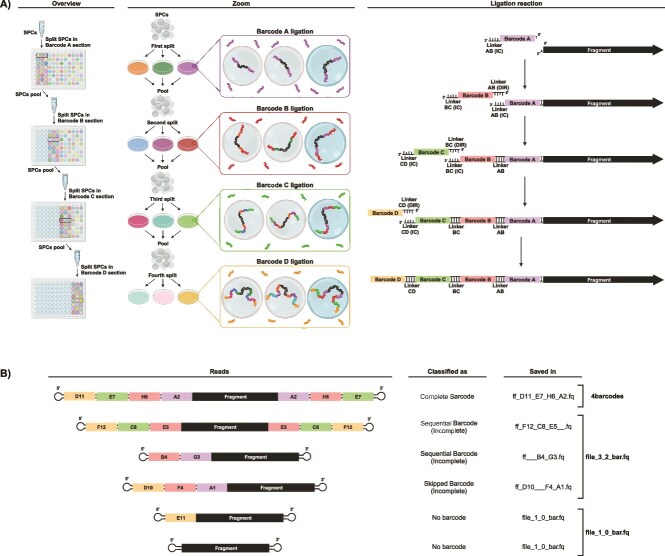
Processing and results of barcoding protocol. (A) **SAG in capsules labeled by split-pool barcoding process.** After whole genome amplification, capsules are splitted into a 24-well section of a plate, where each well has a unique 8-base-pair barcode that binds to the amplified DNA. Following the ligation reaction, the contents are pooled in a new 1.5 ml tube to continue the process in the next section. Three levels of detail are represented: An overview of the protocol that outlines the workflow; a zoom view, that highlights the track of a single capsule; and the ligation reaction which depicts the mechanism of barcode binding. The barcode A ligation depends on a T, incorporated in a prior DNA end-preparation reaction, while subsequent barcodes (B, C, and D) bind through complementary linker interactions. (B) **Possible barcoding outcomes and their processing by CleanBar.** Reads with complete D-C-B-A barcode strings on one end are always saved in a .fastq file named after the four barcodes, even if the string on the other end is missing or incomplete. Reads containing two or three barcodes (either sequential or with skipped positions) are saved in separate .fastq files named accordingly, with the missing position replaced by three underscores (___) and also in a combined file ff_3_2_bar.fq. Reads with only one or no barcodes are grouped in the file named ff_1_0_bar.fq. Created with BioRender.

Split-and-pool barcoding has emerged as an effective alternative to make microbial single-cell omics more accessible for smaller laboratories, reaching the flexibility and high throughput of bulk meta-omics. Atrandi (Vilnius, Lithuania) has commercialized a bench-top-sized microfluidics device, in which individual cells are encapsulated within semipermeable hydrogel capsules, named semi-permeable capsules (SPCs), featuring small pores that allow for the customizable addition and removal of reagents for applications such as DNA extraction and whole-genome amplification, within a single tube [18]. The platform uses split-and-pool barcoding in a 96-well plate format to assign unique barcodes to each capsule by pooling and ligating new barcodes in successive rounds (Fig. 1A). The Atrandi barcoding plate is divided into four three-column sections (A, B, C, and D). First, the encapsulated single-amplified genomes (SAGs) are distributed among the 24 wells in section A, where each well contains a specific barcode for ligation. After the ligation in section A is complete, all capsules are pooled and redistributed to section B for a new round of barcoding, and this procedure repeats through sections C and D. This four-round barcoding process generates unique 44-bp barcodes composed of four 8-bp sequences interspersed by 4-bp linkers. Finally, the barcoded molecules are pooled for sequencing and subsequently de-multiplexed to assign reads back to individual cells (Fig. 1A). The Atrandi platform can potentially generate 331 776 unique barcode combinations (24^4^), significantly exceeding the 10 000 single cells that can be labeled by one kit.

The split-and-pool barcoding approach is not exclusive to microbial single-cell genomics. For instance, Parse Biosciences (USA) has commercialized a split-and-pool barcoding method for eukaryotic transcriptomics, SPLiT-seq, which operates in a 96-well plate format and eliminates the need for large, specialized microfluidics equipment [19]. Rather than encapsulating cells, SPLiT-seq converts them into semipermeable “capsules” through a specialized fixation protocol. Given the cost-effectiveness and high-throughput capacity of split-and-pool barcoding, it is likely that more platforms employing this technique will emerge in the near future.

Most single-cell platforms provide their software for barcode detection and subsequent demultiplexing of reads, such as Cell Ranger for 10X Genomics, or VITAseer for M20 Genomics. Others, like Parse Biosciences, offer a pipeline rather than standalone software for processing and demultiplexing single-cell data. However, researchers often explore novel single-cell omics applications, which require greater bioinformatic flexibility and drive the need for custom computational tools. Examples include BLAZE, designed for demultiplexing 10X Genomics barcodes on the Oxford Nanopore platform [20], and STARsolo for SPLiT-seq [21]. To our knowledge, no flexible, open-source tool currently exists for detecting and demultiplexing reads labeled by repeated ligation of barcodes in a split-and-pool manner, as employed by the Atrandi platform. For this purpose, we introduce CleanBar, a program originally designed to process reads from the Atrandi platform, yet adaptable to a wide range of barcode configurations interspersed with any type of linker.

Detecting reads labeled by split-and-pool barcoding presents several challenges. In large microfluidics instruments used for eukaryotic single-cell transcriptomics, such as those from 10X Genomics, SeekGene or M20 Genomics, long barcodes are directly added to nucleic acids in a ready-to-use format. By contrast, in split-and-pool barcoding, barcodes are constructed as sequential strings. Although the linkers facilitating ligation are designed to have a fixed length (4 bp in Atrandi, 12 bp in Parse Biosciences), occasional minor errors during ligation can cause shifts in the position of subsequent barcodes, potentially failing to detect all downstream barcodes (Fig. 1B). Another complication may involve locating the first barcode, particularly in barcoding platforms that do not involve standardized sequencing adapters nor poly-T sequences to delimit the barcode position, or when sequencing adapters are not fully trimmed.

In this study, we describe the CleanBar software and demonstrate its flexibility using reads barcoded with the Atrandi kit and sequenced on the PacBio platform. Although the Atrandi kit was initially designed for compatibility with the Illumina sequencing platform, the rapid growth of long-read sequencing technologies in recent years prompted us to adapt the protocol for PacBio. PacBio sequencing employs adapters to circularize DNA, enabling error correction through repeated sequencing of the same fragment [22]. In the consensus sequence, these hairpin adapters can be split at any sequence position, leading to varying adapter lengths preceding the biological sequence [23]. Consequently, researchers working with raw reads may encounter challenges in identifying the initial barcode position, particularly if sequencing adapters are not fully removed. Here, however, we demonstrate that CleanBar efficiently detects barcode strings, regardless of their starting position. As a proof of concept, we applied CleanBar to analyze phage-host interactions at single-cell resolution in a *Klebsiella* model system.

## Material and methods

### CleanBar functionality

CleanBar is written in the C programming language, which is compiled automatically by “prepare.sh” script prior to use. It is distributed under a permissive MIT-style license, providing a fast, lightweight, and broadly accessible solution for barcode detection across a range of laboratory settings. CleanBar is applied to a FASTQ file resulting from sequencing, which must be quality-filtered by the user. It also requires a user-defined input text file (barcodes.txt), which should include labels and sequences for all barcode sets in the order, in which they were ligated in the laboratory—in our case, barcodes from sections D, C, B, and A of the Atrandi barcoding plate (Supplementary Fig. S1). The program reads the input FASTQ file and scans each read for the presence of barcode sequences using exact string matching, without allowing for mismatches or indels. It initiates its search with barcode D, as this was the last barcode added to the string. With the -l flag, users can define the length of the initial sequence region where barcodes will be searched. Defining the search length helps prevent the misclassification of non-barcoded reads, as 8 bp barcode-like sequences can incidentally appear within the natural DNA. The predefined search length may exceed the final barcode string length (44 bp in Atrandi, corresponding to 4 × 8 bp barcodes and 3 × 4 bp linkers) since the user might want to account for the untrimmed sequencing adapters preceding the barcode string, additional nucleotides needed to connect the DNA fragment with the first barcode, and potential minor errors in barcode string formation. Barcodes should always be followed by a 4 bp linker; however, small ligation errors can shift the position of the following nucleotide. To account for this, after detection of the barcode D, CleanBar begins searching for a sequence corresponding to barcode C starting from the last nucleotide of the identified barcode D, followed by a search for barcode B from the end of barcode C, and finally for barcode A from the last nucleotide of barcode B. Within each barcode set, CleanBar selects the barcode detected at the closest distance from the preceding barcode. Additionally, the program calculates the distances between the detected barcodes, corresponding to the linker length.

**Figure 2:**
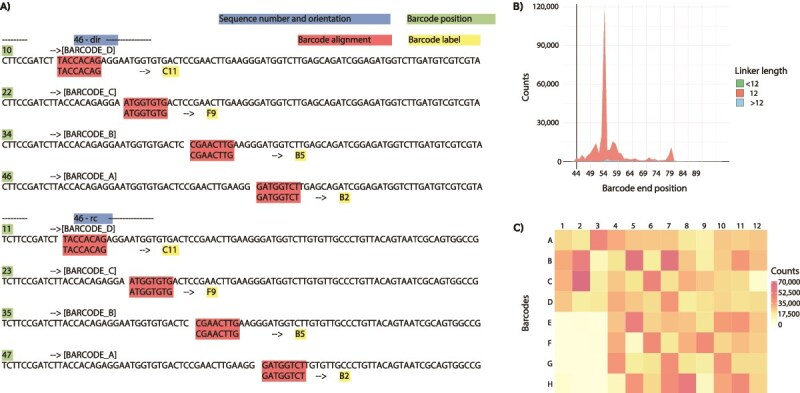
CleanBar barcode detection results. (A) **CleanBar screen output.** Example of a sequence (number 46) showing exact alignment of each of the four barcode groups (D, C, B, A) in both direct (dir) and reverse complement (rc) orientations. The output displays the barcode position, barcode exact alignment, and its label. (B) **Linkers length.** Number of linkers with lengths equal to 12 or shorter or longer than 12 in relation to the barcode end position. Nearly all barcodes have a length of 12. Barcodes detected further from the beginning of the sequence do not have longer linkers between them. (C) **Total read counts per barcode.** Number of reads with a given barcode detected at a given position of the plate.

CleanBar searches for barcodes from both ends of the sequence (Fig. 2A). The barcode string found at one or both ends is trimmed, and the sequence is saved in a FASTQ file with a name corresponding to the identified barcodes. CleanBar then searches the reverse complementary sequence. If a consecutive D-C-B-A barcode sequence is detected in the reverse complementary sequence, the read is appended to the FASTQ file named after the barcodes identified in the direct sequence search. If a file with this name does not yet exist, a new FASTQ file is created. Each sequence will appear in the resulting FASTQ file only once, regardless of whether the barcode string was found on both ends of the sequence or only one.

Ideally, each read should contain four consecutive barcodes in the order D-C-B-A. However, due to occasional ligation failures, this is not always achieved (Fig. 1B). Although incomplete barcode strings do not provide single-cell resolution, some users may still wish to retain reads containing two or three barcodes for further analysis, particularly when designing strategies to optimize laboratory procedures. If no barcode from set D is detected, CleanBar restarts the search with set C. If no barcode C is found, it searches for the B barcodes at the beginning of the read. As a result, FASTQ files are generated with either three or two barcode names, with the missing barcode label replaced by a double underscore string (`__'). Importantly, complete barcode strings have priority in determining the naming and saving of FASTQ files; therefore, sequences with incomplete barcode strings (two or three barcodes) are saved to the corresponding FASTQ file only if no complete D-C-B-A barcode string is detected at the opposite end of the sequence. In exceptional cases, when a read contains different barcode strings on each end (0.4% of reads with complete barcode strings in this experimental case), CleanBar saves the sequence in a FASTQ file with the name derived from the barcode string in the direct sequence. CleanBar also detects barcode strings in which one or two barcode sets were skipped due to ligation errors (Fig. 1B).

Reads with only one barcode are not saved in separate FASTQ files, as any 8 bp sequence could naturally occur in DNA (at probability of 0.25%), providing no definitive evidence that the match results from the barcoding reaction. Instead, reads with only one detected barcode are grouped with reads containing no barcodes in a separate file, <filename>_0_1.fastq. Although sequences in this file lack single-cell resolution, they should not be discarded, as they can still support bulk sequence analyses similar to those in conventional metagenomics.

CleanBar is compatible with any barcode combination. Users only need to update the barcode sequences in the barcodes.txt file and specify the corresponding parameters using the -bn, -bs, -ls, and -l flags. While Atrandi platforms use four sets of 24 barcodes, each eight nucleotides long, other split-and-pool strategies may involve up to 96 barcodes per group and barcodes of different lengths. The number of barcodes per group can be set with the -bn flag, the barcode length with -bs, and the expected linker length with -ls. These settings may also affect the search window at the start and end of each read, which can be adjusted using the -l flag.

CleanBar generates two output text files with statistical information. The tab-separated file <file>_summary.txt lists the barcodes found in both, direct and reverse complement strings for each sequence (Supplementary Fig. S2). The file <file>_stats.txt provides the file name in which each sequence was stored, the position of the last detected barcode, and the lengths of linkers between barcodes (Supplementary Fig. S3). Together, these output files make CleanBar an invaluable tool for evaluating barcoding accuracy and refining laboratory workflows.

CleanBar is compatible with all major operating systems. It runs sequentially on a single processor, as each read must be written to an output file based on its barcode combination, which is a task that cannot be parallelized without risking file access conflicts. To evaluate CleanBar’s performance, we simulated PacBio runs of varying sizes by generating 10 FASTQ files containing between 100 000 and 100 million reads, created by subsampling and replicating reads from our pilot sequencing run. Benchmarking was performed in triplicate on a Linux system with an Intel® Xeon® Silver 4214 CPU @ 2.20 GHz, 384 GB RAM, and a 22 TB magnetic disk. For each file, we recorded the average processing time and number of reads processed per second, providing a basis for assessing CleanBar’s performance across input sizes.

### Pilot testing of phage-bacteria interactions

Our pilot experiment was designed to leverage microbial single-cell genomics to detect active phage infections within individual cells and to differentiate bacteria at the strain level, addressing major challenges in conventional bulk metagenomics [8]. We used two *Klebsiella pneumoniae* strains (Mich61 and 2069/49; capsular types K15 and K16) and two *Klebsiella pasteurii* strains (5725y and 6177; capsular types K29 and K41), each infected with a compatible phage of podovirus, siphovirus, or myovirus morphology (Supplementary Table S1) [24]. Throughout the manuscript, the labels K15, K16, K29, and K41 refer to both the bacterial strains and their corresponding phages. As an internal control for nonspecific attachment, we spiked the four infecting phages with a negative control *Xanthomonas* phage, DoCa5, which cannot infect *Klebsiella* [25]. Stationary-phase bacterial cultures (1 mL; ~10^9^ CFU) were centrifuged, and the cell pellets resuspended in 1.4 mL of a phage mixture (phage X + DoCa5), adjusted to a minimum multiplicity of infection (MOI) of 1 for each phage (Supplementary Methods). Control tubes were prepared similarly, replacing bacteria with LB (phage control) or omitting phages (bacterial control). Tubes were incubated at 37°C for 10 min with shaking (750 rpm), then transferred to 4°C. Tubes were then centrifuged (8000 × g, 3 min), and the supernatants were plated to quantify unabsorbed phages, using the phage control as a reference. Adsorption efficiency for the experimental phages ranged from 88.69% to 99.99% (Supplementary Fig. S4A). Pellets were subsequently washed three times with 1× PBS, and the final supernatants were plated to confirm a substantial reduction of free phages. Washed cells were diluted and plated to estimate viable cell counts, which were compared to the bacterial control to assess phage-induced viability loss, ranging from 91.54% to 99.86% (Supplementary Fig. S4B).

The infected cultures were fixed by methacarn and fluorescence-activated cell sorter (FACS) BD Aria Fusion was used to collect exactly 15 625 cells from each culture, with sorting gates set strictly to particles of bacterial size. According to the Atrandi Flux encapsulation protocol (version 6, 15/11/2023; document DGPM02323108001), this combined set of 62 500 cells (15 625 × 4) maximizes platform capacity, generating up to 10 000 barcoded capsules with only 8% containing multiplets (capsules with multiple cells). The encapsulated cells were lysed and DNA was amplified and barcoded using the Single-Microbe DNA Barcoding kit from Atrandi (ref: CKP-BARK1, version 3 3/11/2023, document: DGPM02323198001), where the whole genome amplification (WGA) step prolonged from 25 min to 60 min to produce longer fragments suitable for PacBio sequencing (details in Supplementary Methods). To assess the natural proportion of barcoded reads, we deliberately avoided PCR-based enrichment of ligated sequencing adapters (Supplementary Methods)—a step included in the Atrandi protocol to aid library preparation and commonly used across sequencing platforms to address this issue [26]. The pooled DNA from the barcoded single-cells was processed by the PacBio SMRTbell v3.0 library preparation protocol (document: 102-166-600 REV02), in which we purposely omitted the Megaruptor 3 fragmentation step, since the expected DNA fragment size produced by WGA falls within the range specified by this library preparation protocol.

The sample was sequenced on the PacBio Sequel II platform with the Sequel II Sequencing Kit 2.0. HiFi PacBio reads were generated using SMRT Link v13.0.0.207600, which includes adapter removal and quality control. Additional quality checks were performed using FastQC v0.12.1 [27] and fastpLong v0.2.2. [28]. The reads were subsequently processed by CleanBar, with the default search length of 88 nucleotides (flag -l 88). The resulting FASTQ files, including those with complete D-C-B-A barcode strings as well as bulk files containing incomplete or absent barcodes, were mapped using bwa-mem v.0.7.17 [29] to a combined set of reference genomes of the four Klebsiella strains, their phages, and the negative control phage, and processed with samtools v.1.16.1 and the -depth flag [30] to calculate read coverage at each position, ensuring each read was counted only once at its highest-quality alignment.

Additionally, we focused on calculating the phage-host genome coverage ratio at the single-cell level. Using SPAdes v.3.15.5 with the -sc flag specific for single-cell assembly [32], we generated separate assemblies for 1916 FASTQ files corresponding to capsules with detectable host-phage pairs containing at least five reads. The resulting contigs were aligned to the phage and host genomes using BLAST v2.14 [33], and contig coverage information generated by SPAdes was used to calculate the average contig coverage for host and phage genomes within each single cell. These values were then used to determine the phage-to-host coverage ratio.

## Results

### Detection of barcodes

WGA yielded 0.134 μg of DNA, with a peak fragment size between 5 063 and 5 875 bp (Supplementary Fig. S5). Sequencing generated 2.3 million reads with an average quality score of 92, and additional quality checks using fastplong confirmed the sequence quality reported by SMRT Link (Supplementary Fig. S6). Of these, CleanBar identified 262 285 reads (11.20%) with complete D-C-B-A barcode strings on one or both ends. Cases where a read contained different barcode strings on each end were observed in only 0.4% of the reads with complete barcode strings in this pilot experiment, and these were excluded from the single-cell analysis. Incomplete but correctly ordered barcode strings, such as D-C-B or B-A, were slightly more frequent, accounting for 13.62% and 3.82% of total reads, respectively. Skipped barcode strings (e.g. D-B-A or C-A) were rare, appearing in only 2.25% of reads with barcodes confirmed on both. As expected, the majority of reads in our pilot run (71.37%) contained only one or no detectable barcode, as PCR-based enrichment for barcode-ligated fragments was intentionally omitted to assess the natural abundance of barcode-free fragments. Most detected linkers were the correct 4 bp length (98.48%), with shorter (3 bp, 0.65%), and longer (5 bp, 0.54%) linkers being less common; linkers of other lengths accounted for only 0.33%.

Although the PacBio reads were trimmed using SMRTLink software v13.0.0.207600, they still contained residues of the sequencing adapters, as evidenced by the majority of complete barcode strings terminating at 54 bp, slightly beyond the expected 44 bp (Fig. 2B). Accurate barcode detection was confirmed even in longer strings, as indicated by the correct linker lengths between barcodes (3 × 4 bp), which accounted for 94.04% of linker lengths across all tested sequence lengths (Fig. 2B).

Individual barcodes in our pilot run showed similar frequencies, except for those from set A in positions E1 to H3, which appeared less frequently (Fig. 2C). The absence of A barcodes corresponds to the higher frequency of incomplete barcodes, as evidenced by D-C-B barcodes appearing 104 times more frequently than C-B-A (based on reads with barcodes confirmed at both sequence ends). This highlights the utility of the CleanBar program in generating barcode statistics that can help optimize laboratory procedures.

The benchmarking of CleanBar with 10 FASTQ files of varying sizes on a benchtop computer confirmed that it is a lightweight and scalable tool for high-throughput sequence processing (Supplementary Fig. S7). For input files containing between 100 000 and 2 million PacBio reads, the processing speed averaged ~8 000 reads per second, with the 2-million-read file completing in approximately 4 min. However, performance declined for input sizes above 5 million reads; the largest file, containing 100 million reads (770 Gbp), was processed at a reduced rate of ~1 200 reads per second, requiring around 24 h to complete.

### Phage bacteria interactions

In total, 15 113 demultiplexed FASTQ files aligned to the reference genome set of *Klebsiella* strains and their phages with a quality score > 60. While this number exceeds the 10 000 files expected based on the Atrandi encapsulation protocol, it is consistent with the anticipated efficiency of the barcoding system. Also, the proportion of true multiplets obtained (6%) was lower than the expected 8%, highlighting the high barcoding efficiency. The files contained an average of 14.1 ± 29.55 reads (range: 1–533, median: 3), which was insufficient to cover the entire bacterial genomes. However, PacBio long-read sequencing enabled clear differentiation of bacterial strains at the read level, as evidenced by only 0.13% of reads with quality scores >60 aligning to more than one bacterial genome.

**Figure 3:**
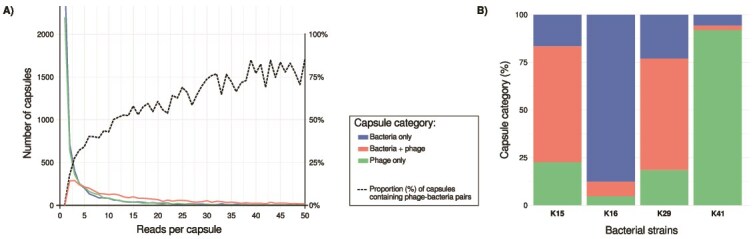
Statistics of capsule content. (A) **Capsule content distribution.** The colored lines represent the number of capsules with bacteria-only, phage-bacteria pair, or phage-only in relation to the reads contained in a capsule (x-axis). With a starting point of five readings per capsule, the number of capsules containing phage-bacteria pairs exceeds those in all other categories. In capsules containing more than five reads, the number of capsules with phage-bacteria pairs is higher than in the other categories. The dashed line shows the proportion (%) of capsules containing phage-bacteria pairs. (B) **Proportion of capsule content by strain.** Proportion (%) of capsules with bacteria-only, phage-bacteria, or phage-only reads within the four Klebsiella spp. strains.

**Figure 4:**
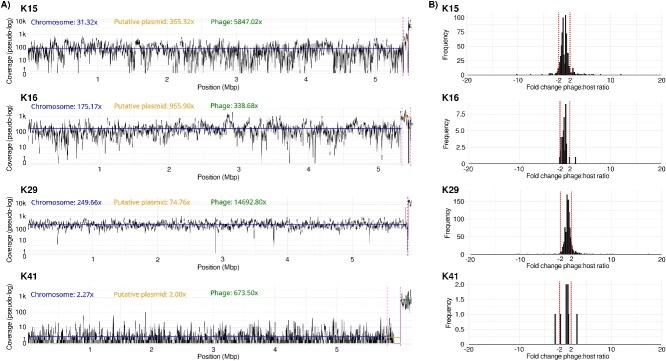
Proportion of reads corresponding to phages and their host bacteria. (A) **Contigs coverage by bulk sequences.** Coverage is represented for each nucleotide position in bacterial chromosomes, putative plasmids, and phage genomes for the four phage-bacteria pairs, based on mapping of reads without barcodes (bulk approach). The three segments (chromosome, plasmid and phage) are separated by vertical dashed lines. Average coverage values for each category are shown in the top-right corner of each plot, and visualized by horizontal lines of the same color. Coverage values were transformed using pseudo-logarithm to enable logarithmic representation while preserving zero values. (B) **Phage-host coverage ratio per capsule.** Ratio is expressed as fold-change values comparing host and phage coverage in each capsule. The single-cell data are shown separately for each bacterial strain. The area within the vertical dashed lines indicates capsules in which phages and their hosts showed similar coverage. The values within the vertical dashed lines indicate similar coverage of phages and their hosts. Negative values indicate higher bacterial coverage than phage coverage, while positive values indicate the reverse.

Phage infection was detectable even in capsules with only two reads, with 7.99% (102 capsules) showing phage-bacteria pairs, highlighting the utility of this approach for studying phage–bacteria interactions even at shallow sequencing depth per cell. In capsules with as few as five reads (629 capsules), the proportion containing host-phage pairs (38.84%, 216 capsules) exceeded that of bacteria-only capsules (24.69%, 201 capsules), demonstrating that even shallow genome coverage can reliably identify infected cells (Fig. 3A). Overall, 77.22% of the 6 773 capsules with at least five reads contained detectable phage sequences, a proportion consistent with plaque assay results (Supplementary Fig. S4). This detected rate increased with read depth, reaching 84.13% among the 1 109 capsules that contained at least 50 reads. Both plaque assays and single-cell sequencing analyses indicated that strain K16 had the lowest infection rate among the four strains (Fig. 3B**,**  Supplementary Fig. S4). However, the proportion of infected K16 cells observed at the single-cell level (7.55% out of 794 capsules%) was considerably lower than the 91.54 ± 59.70% infection rate measured in the laboratory, a discrepancy likely caused by an inaccurate live-to-dead cell ratio estimation that does not account for potential enrichment of cells that developed resistance to the phages during incubation or died for other reasons. The low infection rate in K16 was also evident at the bulk scale (using FASTQ files containing reads with incomplete or missing barcodes), where K16 phage genome coverage was only 1.9× higher than that of the host genome, indicating limited phage replication at the population scale (Fig. 4A).

The assay appears to have identified signatures consistent with phage infections with high burst sizes, as indicated by the absence of host DNA in 19.26% of capsules with more than five reads. Given the strict FACS sorting settings, which selectively collect bacterial-sized particles and minimize phage-only capsules, the absence of host DNA suggests the presence of hundreds of phage particles within a single cell. This was particularly evident in *K. pneumoniae* K41, where phage-only capsules were 40x more prevalent than capsules containing phage-bacteria pairs (Fig. 4B). The high portion of K41 was also evident on the bulk scale, where the coverage of the K41 phage obtained by mapping the reads with incomplete or absent barcodes was 297x higher than the coverage of the K41 host genome (Fig. 4A).

Taking advantage of the uniform coverage in our assay (Fig. 4A), we estimated phage copy numbers by comparing coverages of bacterial and phage contigs in 1 916 capsules that contained at least five reads per FASTQ file. Overall, host and phage coverages were similar (within a 2× range) in 88%–95% of capsules containing both phages and hosts, with the highest detected fold-changes in the phage-to-host and host-to-phage ratios being also similar: 17 and 20, respectively (Fig. 4B). This result is unsurprising, as in our dataset the extremely active phage replication within a cell is rather indicated by the absence of host DNA, as observed especially in the K41 strain, in which 94.38% of capsules with more than five reads did not contain host DNA (Fig. 3B).

Importantly, the internal negative control phage DoCa5 was detected in only 80 capsules and showed no bias toward any of the strains, being detected in only 0.47%, 0.23%, 0.42%, and 0.56% of the total capsules assigned to strains K15, K16, K29, and K41, respectively. Although the DoCa5 phage appeared to adsorb to the K16 strain in laboratory assays (Supplementary Fig. S4), it was detected minimally in the sequence data, suggesting that its adsorption was reversible, likely detaching during culture media removal by centrifugation or flow cytometry.

## Discussion

We originally developed CleanBar to demultiplex microbial single-cell genomics data from split-and-pool barcoding on the Atrandi platform; however, its versatility allows detection of any barcodes added through sequential ligations, regardless of their initial positions or linker lengths, making it applicable to diverse barcoding and sequencing platforms. Additionally, its barcode detection algorithm is independent of sequencing adapter sequences, allowing barcode recovery even when adapters are not properly trimmed, and enabling compatibility with a range of sequencing technologies, including long-read platforms currently driving advances in metagenomics [33]. CleanBar processes one sequence at a time and runs on a single processor, resulting in very low memory usage, typically less than 1 GB of RAM is sufficient. This makes the tool suitable for execution on standard laptops and low-specification desktop computers. The primary hardware constraint is disk space, which should be approximately 1.2x the size of the input FASTQ file to accommodate all generated output files (e.g. 120 GB for a 100 GB input file). Performance may decline slightly with very large input files (e.g. >5 million reads), likely due to the increasing size of the demultiplexed FASTQ files, which must be updated with each new read added. Overall, CleanBar is a practical and accessible solution for a wide range of laboratories, regardless of their computing infrastructure.

We demonstrated the application of CleanBar using bacterial cultures of closely related *Klebsiella* strains infected with phages. This assay enabled us to benchmark the ability of single-cell sequencing to resolve phage-host interactions at a high-throughput scale, while overcoming the core limitation of bulk metagenomics—its population-averaged nature, which precludes determining whether phage and host sequences detected in the same sample truly co-occurred within individual cells. Although several bioinformatic tools can infer phage-host associations in bulk metagenomes using markers such as CRISPR spacers or phage-encoded bacterial tRNAs, these signals reflect past infections and offer limited insight into current interactions, particularly given the rapid evolution of bacterial resistance mechanisms [34]. Proximity ligation methods, such as Hi-C, attempt to approximate single-cell resolution by detecting physical DNA associations within bulk samples and have proven effective for identifying phage-host interactions [35, 36]. However, Hi-C methods rely on coverage-based inference, requiring deep sequencing and high-quality reference genomes, which can be difficult to obtain from complex microbiomes, and their results may be affected by ligation bias [37, 38]. In contrast, single-cell genomics preserves the native cellular content, which is particularly valuable for revealing broad-host-range phages or co-infections by multiple phages within a single host, that are largely invisible to bulk methods.

It is also important to highlight the laboratory efficiency of the split-and-pool barcoding approach in single-cell genomics: the single pilot experiment in this study produced a data volume equivalent to eight months of conventional single-cell genomics while reducing laboratory expenses by 100-fold (excluding human labor). This substantial gain in efficiency is largely due to the complete elimination of FACS-based cell sorting into 96- or 384-well plates and qPCR-based monitoring of the WGA, which are the steps that typically constrain throughput to a single plate per day. In support of this, we obtained 15 113 demultiplexed FASTQ files from a single barcoding experiment, exceeding the ~10 000 files expected based on the Atrandi encapsulation protocol, which likely reflects the precision of manual capsule retrieval.

WGA, particularly its most commonly used variant, multiple displacement amplification, is often criticized for producing sparse genome coverage with regions over-amplified thousands of times [17, 31, 39–43]. However, this issue was not observed with the Atrandi WGA kit in our pilot run, likely due to its optimized chemistry, which enables estimation of phage copy numbers within single cells. Using this approach, we uncovered population-level heterogeneity in phage infection, potentially reflecting physiological variability within clonal populations [44]. Similar heterogeneity has been reported in previous studies, typically investigated using low-throughput 96-well plate assays based on phage culturing or qPCR, which are limited to cultured bacteria, or microscopy-based methods such as PhageFISH, which can also be applied to uncultured bacteria but remain low-throughput [45–47]. Although culture-, qPCR-, and microscopy-based methods provide valuable information on phage burst size at the single-cell level, DNA sequencing offers insight into another important aspect of phage infection heterogeneity—the variation in phage insertion sites within bacterial genomes [48], which can be further explored using single-cell genomics.

In our pilot sequencing run, we generated 2.3 million PacBio reads (8.9 Gbp in total), providing approximately 1 600x coverage of the *Klebsiella* genome. While this coverage is insufficient to assemble complete genomes from the over 15 000 captured single cells, it reflects a common constraint in single-cell genomics: although WGA typically yields enough DNA to recover full genomes from all bacterial cells in the assay, actual sequencing depth is often limited by budget rather than DNA availability. Nevertheless, even with relatively low coverage, our assay successfully detected host-phage pairs, including in single-cells represented by as few as five reads, suggesting it is a feasible approach for detecting phage infections in natural environments where phages are present at low copy numbers, such as environmental biofilms or the human gut [13, 49]. In environments where phages exhibit large burst sizes, such as seawater [50], shallow sequencing may result in capsules yielding no host DNA, as exemplified by phage K41, which likely replicated to such an extent within a cell that the host DNA became undetectable. In these cases, as demonstrated in our study, FACS prior to encapsulation can select intact bacterial cells and eliminate free virions, which would otherwise be encapsulated alongside other single particles present in the sample. In order to identify bacterial hosts of phages with large burst sizes, a deeper sequencing coverage would be recommended.

The target sequencing depth should be tailored to the specific research questions. In many cases, especially when analyses can be performed by mapping reads to existing reference genomes, recovering complete genomes from 10 000 to 15 000 bacterial single-cells in a single assay is unnecessary. When studying novel microbial communities without available reference genomes and under budget constraints, researchers can treat Atrandi reads as conventional bulk metagenomic data by removing the barcodes and assembling metagenome-assembled genomes (MAGs) [51]. The original barcoded reads can then be mapped back to the MAGs, enabling strain-level resolution and addressing a range of biological questions.

In our pilot project, we focused on evaluating the capability of the Atrandi assay to discriminate bacterial strains and to identify cells infected by phages, both of which are essential for analyzing host-phage interactions in environmental samples. The results showed that only a minor proportion of reads (0.13%) aligned to multiple *Klebsiella* genomes, likely reflecting highly conserved genomic regions shared among the strains. Minimal adsorption of the nonspecific control phage suggests that accidental attachment is unlikely to be a major concern in this assay, though it should still be considered when applied to more complex systems. Altogether, the results demonstrate that split-and-pool barcoding is highly suited to advancing microbial ecology across diverse contexts, from ecosystem dynamics to microbial evolution. The thousands of genomes of novel microbes linked to their mobile genetic elements that this assay can generate will provide a foundation for microbial single-cell transcriptomics, which is likely to expand to the scale of its eukaryotic counterpart in the near future [7, 52].

## Conclusions

Our study demonstrates the combined strength of the Atrandi microfluidics platform, PacBio long-read sequencing, and CleanBar demultiplexing in detecting phage-host associations at single-cell resolution, including patterns suggestive of ongoing infection. CleanBar, presented here, is a flexible barcode detection algorithm that runs on a standard benchtop computer and is adaptable to various split-and-pool barcoding schemes and sequencing platforms. It also provides useful statistics for optimizing laboratory procedures. This integrated system offers a substantial increase in throughput and cost-efficiency, addressing traditional bottlenecks in single-cell genomics, which are often limited to processing one plate per day, and achieves throughput comparable to traditional metagenomics while maintaining single-cell resolution. The improved chemistry of the Atrandi WGA kit yields uniform genome coverage, enabling estimation of phage burst size at the single-cell level, which revealed heterogeneity in phage infection within clonal bacterial populations. We also showed that the assay effectively distinguishes between bacterial strains and prevents non-specific phage attachment, supporting its suitability for high-throughput assessment of phage-host interactions in complex microbial communities across diverse environments.

## Supplementary Material

SupplementaryFigureS1_ycaf134

SupplementaryFigureS2_ycaf134

SupplementaryFigureS3_ycaf134

SupplementaryFigureS4_ycaf134

SupplementaryFigureS5_ycaf134

SupplementaryFigureS6_ycaf134

SupplementaryFigureS7_ycaf134

SupplementaryTableS1_ycaf134

SupplementaryMethods-2_ycaf134

## Data Availability

The sequencing data are available at NCBI with BioProject ID: PRJNA1199928. The CleanBar program source code and test files coming from the same sequence dataset are available at the GitHub repository https://github.com/tbcgit/cleanbar. The complete FASTQ outputs of the CleanBar program (individual FASTQ files containing 4 barcodes and the FASTQ files with 2 or 3 barcodes), as well as the R script for generating raw figures with the corresponding source files are available for download from the repository of University of Valencia https://nuvol.uv.es/owncloud/index.php/s/pSpjXkmx7EW8Oum.
