# Supplementary material for: CleanBar: a versatile demultiplexing tool for split-and-pool barcoding in single-cell omics
Source: ISME Commun. 2025 Aug 1;5(1):ycaf134. doi: 10.1093/ismeco/ycaf134 (PMC12376035; doi:10.1093/ismeco/ycaf134)
Supplement: SupplementaryFigureS1_ycaf134 [file supplementaryfigures1_ycaf134.pdf]

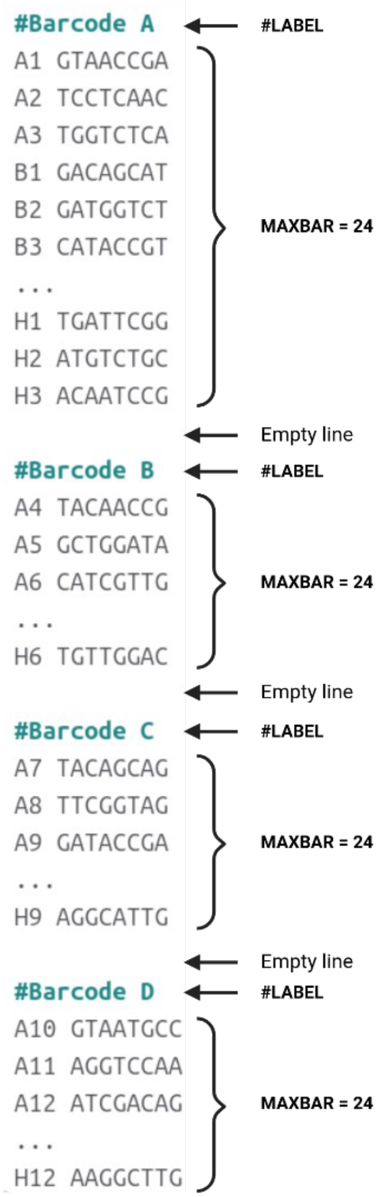

Supplementary Figure S1. Structure of required input file. Example of the user-defined input text file with the barcode sets used in the experiment. For each barcode set, for each group of barcodes, there must be a first label of the group. Then the names of the barcodes and the sequence corresponding to them must be displayed, for up to a maximum of 24 sequences. Each barcode set must be separated by an empty line.
