# Supplementary material for: CleanBar: a versatile demultiplexing tool for split-and-pool barcoding in single-cell omics
Source: ISME Commun. 2025 Aug 1;5(1):ycaf134. doi: 10.1093/ismeco/ycaf134 (PMC12376035; doi:10.1093/ismeco/ycaf134)
Supplement: SupplementaryFigureS2_ycaf134 [file supplementaryfigures2_ycaf134.pdf]

@m64105_240622_213943/16/ccs	Direct	1	...	-1	...	-1	...	-1	...	-1	Rev_com	1	D10	31	G8	43	H5	55	B2	67
@m64105_240622_213943/37/ccs	Direct	2	...	-1	...	-1	...	-1	...	-1	Rev_com	2	...	-1	...	-1	...	-1	...	-1
@m64105_240622_213943/46/ccs	Direct	3	...	-1	...	-1	...	-1	...	-1	Rev_com	3	E12	33	B9	45	E4	57	...	-1
@m64105_240622_213943/54/ccs	Direct	4	C10	18	D9	31	C4	43	...	-1	Rev_com	4	...	-1	...	-1	...	-1	...	-1
@m64105_240622_213943/60/ccs	Direct	5	F12	10	H8	22	G4	34	A3	46	Rev_com	5	...	-1	...	-1	...	-1	...	-1
@m64105_240622_213943/73/ccs	Direct	6	B10	10	B7	22	F6	34	...	-1	Rev_com	6	...	-1	H9	69	...	-1	...	-1
@m64105_240622_213943/21/ccs	Direct	7	...	-1	...	-1	...	-1	...	-1	Rev_com	7	B10	41	...	-1	...	-1	...	-1
@m64105_240622_213943/24/ccs	Direct	8	...	-1	...	-1	...	-1	...	-1	Rev_com	8	...	-1	...	-1	...	-1	...	-1
@m64105_240622_213943/27/ccs	Direct	9	...	-1	...	-1	...	-1	...	-1	Rev_com	9	...	-1	...	-1	...	-1	...	-1
@m64105_240622_213943/13/ccs	Direct	10	...	-1	...	-1	...	-1	...	-1	Rev_com	10	H11	15	...	-1	...	-1	...	-1
@m64105_240622_213943/61/ccs	Direct	11	...	-1	...	-1	...	-1	...	-1	Rev_com	11	...	-1	...	-1	...	-1	...	-1
@m64105_240622_213943/76/ccs	Direct	12	...	-1	...	-1	...	-1	...	-1	Rev_com	12	...	-1	...	-1	...	-1	...	-1
@m64105_240622_213943/85/ccs	Direct	13	...	-1	...	-1	...	-1	...	-1	Rev_com	13	...	-1	...	-1	...	-1	...	-1
@m64105_240622_213943/6/ccs	Direct	14	...	-1	...	-1	A5	46	...	-1	Rev_com	14	G11	10	B8	22	B5	34	C2	46
@m64105_240622_213943/7/ccs	Direct	15	...	-1	...	-1	...	-1	...	-1	Rev_com	15	...	-1	...	-1	...	-1	...	-1

Supplementary Figure S2. Screenshot of <file>_summary.txt. Each row presents (in order) the read name, the orientation of the sequence, the number of the read analyzed, the name of the barcode assigned, and its position in the sequence (in both direct and reverse complementary orientation).
