# Supplementary material for: CleanBar: a versatile demultiplexing tool for split-and-pool barcoding in single-cell omics
Source: ISME Commun. 2025 Aug 1;5(1):ycaf134. doi: 10.1093/ismeco/ycaf134 (PMC12376035; doi:10.1093/ismeco/ycaf134)
Supplement: SupplementaryFigureS3_ycaf134 [file supplementaryfigures3_ycaf134.pdf]

@m64105_240622_213943/16/ccs	1_dir	------	0	(#0)				1_rc	ff_D10_G8_H5_B2.fq	67	(#4)	4	4	4
@m64105_240622_213943/60/ccs	5_dir	ff_F12_H8_G4_A3.fq	46	(#4)	4	4	4	5_rc	------	0	(#0)			
@m64105_240622_213943/6/ccs	14_dir	------	46	(#1)				14_rc	ff_G11_B8_B5_C2.fq	46	(#4)	4	4	4

Supplementary Figure S3. Screenshot of <file>_stats.txt. This file contains the information of the reads with 4 barcode sequences. Each row represents a single read. The columns show 1.) read name, followed by information for the direct sequence: 2.) the number and the orientation of the read; 3.) the FASTQ file named after the barcode sequence; 4.) position of the last nucleotide of the barcode sequence; 5.) number of detected barcodes, 6-8.) the length between barcodes (D-C, C-B, and B-A); which is followed by the same information for the reverse complement sequence (columns 9-15).
