# Supplementary material for: CleanBar: a versatile demultiplexing tool for split-and-pool barcoding in single-cell omics
Source: ISME Commun. 2025 Aug 1;5(1):ycaf134. doi: 10.1093/ismeco/ycaf134 (PMC12376035; doi:10.1093/ismeco/ycaf134)
Supplement: SupplementaryFigureS4_ycaf134 [file supplementaryfigures4_ycaf134.pdf]

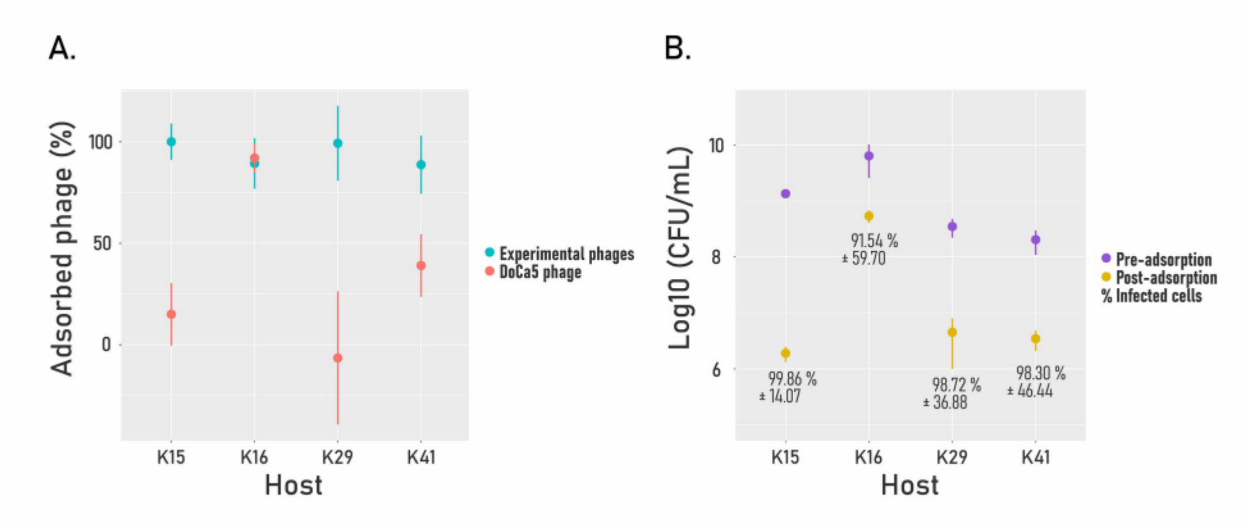

Supplementary Figure S4. Phage plaque assay results. A) Proportion of adsorbed phage. The percentage of phages adsorbed to their respective bacterial host was measured for the experimental phages (K15PH90, K16PH164C3, K29PH164C1, and K41P2) and the negative control phage (DoCa5). The experimental phages exhibited high adsorption efficiency, whereas DoCa5 did not, with the exception of K16. **B)** Reduction in bacterial counts after phage adsorption. A decrease in bacterial concentration after adsorption confirms that the adsorbed phages successfully infected the bacterial cells. Error bars represent the SEM. The proportion of infected cells is presented as percentage (% ± standard error (SE)).
