# Supplementary material for: CleanBar: a versatile demultiplexing tool for split-and-pool barcoding in single-cell omics
Source: ISME Commun. 2025 Aug 1;5(1):ycaf134. doi: 10.1093/ismeco/ycaf134 (PMC12376035; doi:10.1093/ismeco/ycaf134)
Supplement: SupplementaryFigureS5_ycaf134 [file supplementaryfigures5_ycaf134.pdf]

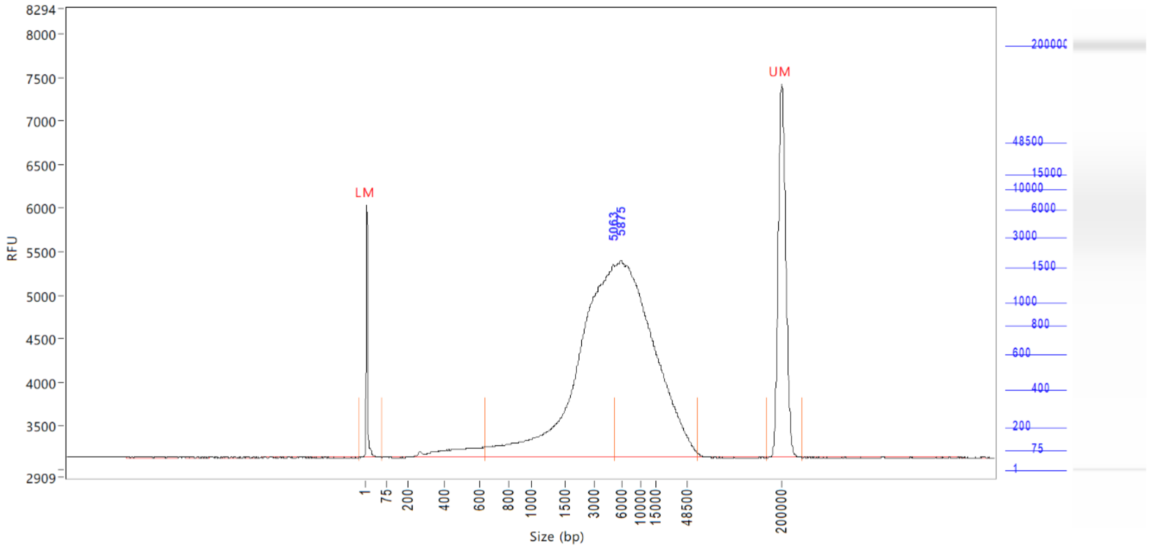

Sample Peak Width (sec): 10	Sample Min Peak Height: 50	Sample Baseline V to V?: Y	Sample Baseline V to V pts: 3
Sample filter: Binomial	Number of points for filter: 3	Sample start region (min): 0	Sample end region (min): 55
Manual baseline start (min): 12	Manual baseline end (min): 53	Marker peak width (sec): 5	Marker min peak height: 500
Marker baseline V to V?: N	Marker baseline V to V points: 3	Lower marker selection: First peak > 500 RFU	Upper marker selection: Last peak > 500 RFU
Ladder size (bp)1, 75, 200, 400, 600, 800, 1000, 1500, 3000, 6000, 10000, 15000, 48500, 200000	Quantification using: Ladder Final concentration (ng/uL): 0.1250 Dilution factor: 12.0	Minimum RFU for data processing: 3	

Supplementary Figure S5. Fragment Analyzer results. Size distribution analysis of resulting genome-amplified fragments.
