# Supplementary material for: CleanBar: a versatile demultiplexing tool for split-and-pool barcoding in single-cell omics
Source: ISME Commun. 2025 Aug 1;5(1):ycaf134. doi: 10.1093/ismeco/ycaf134 (PMC12376035; doi:10.1093/ismeco/ycaf134)
Supplement: SupplementaryFigureS6_ycaf134 [file supplementaryfigures6_ycaf134.pdf]

A)

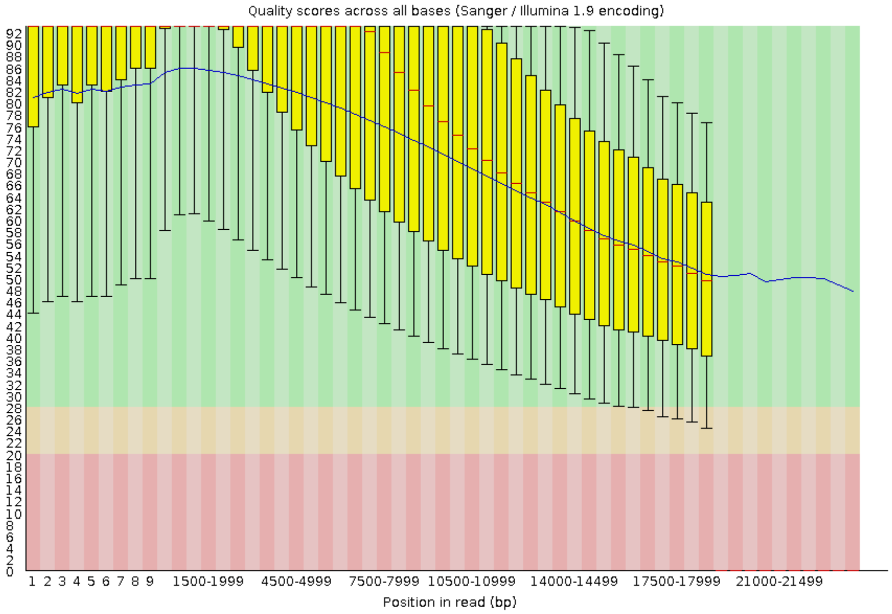

B)

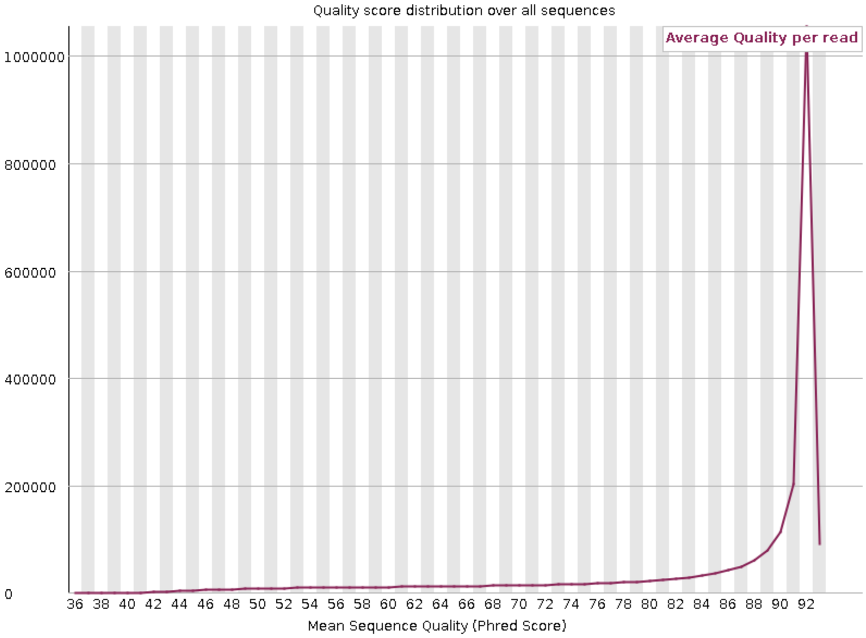

Supplementary Figure S6. FastQC sequence quality analysis results. A) Per base sequence quality. **B)** Per sequence quality scores.
