# Supplementary material for: CleanBar: a versatile demultiplexing tool for split-and-pool barcoding in single-cell omics
Source: ISME Commun. 2025 Aug 1;5(1):ycaf134. doi: 10.1093/ismeco/ycaf134 (PMC12376035; doi:10.1093/ismeco/ycaf134)
Supplement: SupplementaryFigureS7_ycaf134 [file supplementaryfigures7_ycaf134.pdf]

A)

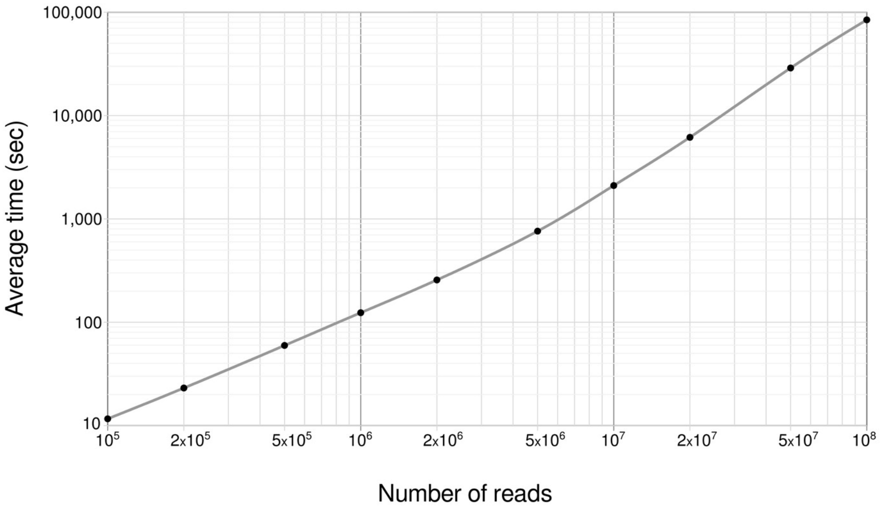

B)

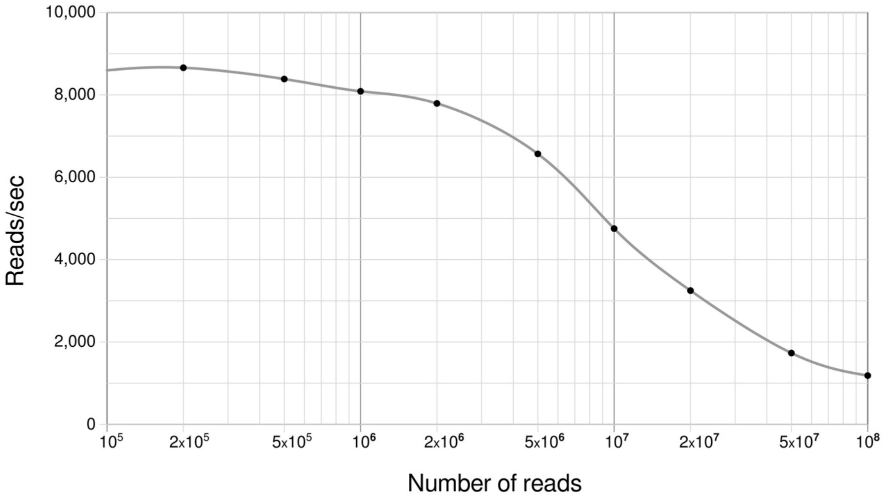

Supplementary Figure S7: Benchmarking CleanBar performance. A) Average analysis time as a function of input read number. **B)** Processing speed (reads per second) relative to input size. Analyses were performed on a system with an Intel® Xeon® Silver 4214 CPU @ 2.20 GHz, 384 GB RAM, and 22 TB of magnetic storage.
