# Supplementary material for: CleanBar: a versatile demultiplexing tool for split-and-pool barcoding in single-cell omics
Source: ISME Commun. 2025 Aug 1;5(1):ycaf134. doi: 10.1093/ismeco/ycaf134 (PMC12376035; doi:10.1093/ismeco/ycaf134)
Supplement: SupplementaryTableS1_ycaf134 [file supplementarytables1_ycaf134.pdf]

Supplementary Table S1: List of bacterial strains and phages used

Bacteria			
**Capsular-type**	**Name**	**Species**	**Reference**
K15	Mich. 61	*K. pneumoniae*	NCTC9135
K16	2069/49	*K. pneumoniae*	NCTC9136
K29	5725y	*K. pasteurii*	NCTC9149
K41	6177	*K. pasteurii*	NCTC9161

Phages								
**Name**	**Complete name**	**Reference**	**Host (Capsular-type)**	**Morphology**	**Genome (bp)**	**Family**	**Subfamily**	**Genus**
K15PH90	vB_Kpn_K15PH90	OY979410	*K. pneumoniae* Mich.61 (K15)	Podovirus	44292	*Autographiviridae*	*Slopekvirinae*	*Drulisvirus*
K16PH164C3	vB_Kpn_K16PH164C3	OY979392	*K. pnuemoniae* 2069/49 (K16)	Podovirus	39283	*Autographiviridae*	*Studiervirinae*	*Przondovirus*
K29PH164C1	vB_Ko_K29PH164C1	OY757094	*K. pasteurii* 5725y (K29)	Siphovirus	52798	*Drexlerviridae*	unclassified	unclassified
K41P2	vB_Ko_K41P2	OY979424	*K. pasteurii* 6177 (K41)	Myovirus	176681	*Straboviridae*	unclassified	*Slopekvirus*
DoCa5*	vB_Xar_IVIA-DoCa5	NC_073026	*Xanthomonas* spp	Siphovirus	56407	*Mesyonzhinoviridae*	*Bradleyvirinae*	unclassified

*Negative control phage
