# Supplementary material for: CleanBar: a versatile demultiplexing tool for split-and-pool barcoding in single-cell omics
Source: ISME Commun. 2025 Aug 1;5(1):ycaf134. doi: 10.1093/ismeco/ycaf134 (PMC12376035; doi:10.1093/ismeco/ycaf134)
Supplement: SupplementaryMethods-2_ycaf134 [file supplementarymethods-2_ycaf134.pdf]

Supplementary Methods

for article:

CleanBar: A Versatile Demultiplexing Tool for Split-and-Pool Barcoding

Vicente Arnau, Alicia Ortiz Maiques, Juan Valero Tebar, Lucas Mora-Quilis, Vaida Kurmauskaite, Lorea Campos Dopazo, Pilar Domingo-Calap, Mária Džunková

Index:

Phage plaque assays ……………………………………………………………………2

Microbial single-cell genomics ………………………………………………………5

1. Phage plaque assays

Phage and Bacterial Stocks:

- Phage stocks were prepared at a minimum concentration of 10^{10} PFU/mL.
- Bacterial stocks were at a minimum concentration of 10^9 CFU/mL.

  Each phage was amplified and plated on its respective bacterial host (e.g., Phage K15PH90 on *K. pneumoniae* strain K15 (Mich. 61); Phage K16PH164C3 on *K. pneumoniae* strain K16 (2069/49), etc.).
  Lysogeny-Broth (LB) supplemented with calcium chloride ($CaCl_2$) at a final concentration of 0.42 g/L was used as a growth medium.

Experimental and control conditions:

For each bacterium and its respective phage, the following conditions were prepared, where "X" represents the experimental bacterium-phage pair:

- **Experimental tube**:
  Bacteria X + Phage X + Phage DoCa5 (final volume (Vf) = 1.4 mL).
- **Phage control tube** *(non-adsorbed free phage control)*:
  Phage X + Phage DoCa5 (Vf = 1.4 mL).
- **Bacterial control tube** *(to compare the number of viable cells related to the experimental tube)*:
  Bacteria X + LB (Vf = 1.4 mL).

Phage adsorption to bacterial cells:

1. We took 1 mL of stationary-phase bacterial culture (containing approximately 10^9 CFU).
2. The culture was centrifuged to pellet the cells, and the supernatant was discarded.
3. The cell pellet was resuspended in 1.4 mL of phage suspension containing both the experimental and control phage (DoCa5), ensuring a minimum MOI of 1 for each phage.
   - For the phage control tube, LB was used instead of bacteria.
   - For the bacterial control tube, no phage was added, resuspending the bacterial cells in LB instead.
4. Tubes were incubated at 37 ºC for 10 minutes with shaking at 750 revolutions per minute (rpm).
5. After incubation, the tubes were kept at 4 ºC for subsequent processing.

6. An aliquot of these tubes was taken for further testing.
   6.1. Tubes were centrifuged at 8,000 x g for 3 minutes and supernatants were plated to determine the percentage of phages adsorbed by comparing the concentration of phages after inoculation with the concentration in the phage control tube **(Supplementary Figure S4a)**.
   6.2. The supernatants were discarded, and the cell pellets were washed with 1x phosphate-buffered saline (PBS). This was repeated two more times.
      - After the final washing step, the supernatants were plated to confirm that the concentration of non-adsorbed free phage was lower than before washing.
   6.3. The washed cultures were further diluted and plated to estimate the number of viable cells. The bacterial control tube was used to compare the number of non-infected cells to those in the experimental tube **(Supplementary Figure S4b).**
7. The remaining 1,050 µL were centrifuged at 8000 x g for 3 minutes, and the supernatants were discarded.
8. The pellets were resuspended in Methacarn (60% methanol, 30% chloroform, 10% glacial acetic acid)
9. The cultures were incubated for 1 hour at room temperature.
10. After incubation, the cultures were washed with 1x PBS.

Phage Adsorption and Infected Cells Estimation

The percentage of adsorbed phage was estimated by comparing the phage concentration in the experimental tube after incubation ($[Phage_{exp})$ to the concentration of phage in the control tube ($[Phage_{Ctl})$ (*section 6.1*). The mean was obtained using at least three technical replicates, with the standard error of the mean (SEM).

The percentage of adsorbed phage was calculated as:

$$\%Adsorption = 100\% - \left(\frac{[Phage_{exp}]x100}{[Phage_{Ctl}]}\right) \pm \Delta z$$

Where Δz represents the error, calculated as:

$$\Delta z = \sqrt{\left(SEM^2{}_{Phage_{exp}} + SEM^2{}_{Phage_{Ctl}}\right)}$$

To estimate the percentage of infected cells the same procedure was followed by comparing the concentration of viable cells in the experimental tube after washing ($[Bacteria_{exp})$ to the concentration of bacteria in the control tube

($[Bacteria_{Ctl}$) (*section 6.3*). The mean was calculated from at least three technical replicates, with the SEM.

The percentage of infected cells was calculated as:

$$\%InfectedCells = 100\% - \left(\frac{[Bacteria_{exp}]x100}{[Bacteria_{Ctl}]}\right) \pm \Delta z$$

Where Δz represents the error, calculated as:

$$\Delta z = \sqrt{\left(SEM^2{}_{Bacteria_{exp}} + SEM^2{}_{Bacteria_{Ctl}}\right)}$$

2. Microbial single-cell genomics

Single-cell encapsulation in Semi-Permeable Capsules (SPCs)

The encapsulation of a 62,500-cell mixture from four samples of methacarn-fixed *Klebsiella* species was conducted using the Atrandi microfluidic device, FLUX, along with its corresponding SPCs Generation Kit (CKP-BARK1, version 6). The process is supported by three reagents that establish the optimal environment for generating and stabilizing semi-permeable capsule formation around individual cells. First, we prepared the working solutions with the following components:

- **Working Core Solution (WCS)** by combining 50 µL of 2x Core Reagent, 1 µL of DTT, 12.5 µL of photoinitiator, and 36.5 µL of the sample.
- **Working Shell Solution (WSS)** by combining 50 µL of 2x Shell Solution and 50 µL of phosphate-buffered saline (PBS) solution

Once prepared, these solutions were loaded into the corresponding chip well, as well as the third required solution, the Capsule Stabilization Oils (CSO), in the next order and volume: 300 µL of CSO, 100 µL of WSS and 100 µL of WCS. The SPC Generation Chip was placed into the FLUX system where the encapsulation reaction and shell crosslinking occurred for half an hour.

After completing the encapsulation run, the generated emulsion was transferred to a new 1.5 mL tube where the emulsion breaking was performed. To the recovered volume of the emulsion with SPCs, we added 500 µL of Capsule Wash Buffer (prepared by diluting 50x Wash Additive to 1x with PBS) and 500 µL of Emulsion Breaker. This mixture was inverted 5-10 times and spinned down with a tabletop centrifuge. Then, the SPCs concentrated at the interface between an aqueous phase on the top and the emulsion breaker solution on the bottom. The SPCs were resuspended in the aqueous layer and transferred to a fresh tube. The resulting capsules were washed four times to remove residual oils by 1-minute centrifugation at 1000 g, removing the supernatant, and resuspending the capsules in 1 mL of 1x Capsule Wash Buffer.

Cell fixation

Although our samples were already fixed with methacarn, the protocol for Single-Microbe DNA Barcoding (version 3) includes a cell fixation process with 100% methanol. For each 100 µL of SPC sample, we added 900 µL of previous cold methanol drop by drop while agitating the capsules in the tube. After the fixation, the sample was stored at -20 ºC for 30 minutes before the cell lysis. Then, the SPCs were washed five times with 1x Wash Buffer as explained in the previous step.

Cell lysis and Whole Genome Amplification (WGA)

Encapsulated cells underwent a lysis process. First, we adjusted the total volume of the SPCs up to 500 µL by adding 1x Wash Buffer. Then, 500 µL of Lysis Buffer, prepared by 2x fresh alkaline Lysis buffer with 100 µL of 1M DTT, was added to the SPC suspension and mixed by vortexing. The treatment lasted 15 minutes while the tube was rotating at room temperature. Finally, the reaction was stopped with 5 washes with 1 mL of Neutralization buffer and 5 more washes with 1x Wash Buffer.

After the lysis process, the protocol proceeds with the Whole Genome Amplification (WGA) reaction. The reaction mix combined 150 µL of SPCs, 97.5 µL of Nuclease-Free Water (NFW), 37.5 µL of 10x WGA Reaction Buffer, 37.5 µL of dNTP Mix, 18.75 µL of Primer Mix, 3.75 µL of 0.1M de DTT, 3.75 µL of 10% Pluronic F-68, 7.5 µL of WGA Enhancer and 18.75 µL of WGA Polymerase. This mixture was distributed into 5 PCR tubes, each contained 75 µL of the mixture. The reaction was performed in a thermocycler set to incubation for 1 hour at a constant temperature of 45 ºC, followed by a final amplification step at 65 ºC for 10 minutes. Following amplification, the content of the five tubes was pooled into the same tube and 3 washed with 1 mL of Wash Buffer. Additionally, in order to confirm the success of the reaction, 1 µl of capsules was stained with 1 µl of 100x dilution of SYTO 9 (Invitrogen, ref: S34854) and analyzed with fluorescent microscopy (Leica Thunder DMI8).

Single-Cell Barcoding (Split-Pool Method)

The debranching and end preparation reactions were performed according to the Single-Microbe DNA Barcoding protocol (version 3). First, we prepared the debranching mixture consisting of 150 µL of SPCs, 30 µL of 10x Debranching Buffer, 15 µL of Debranching Enzyme, and 105 µL of NFW. The reaction was performed on a thermomixer set to 37ºC and 1000 rpm for 1h. Afterwards, three washes with 1 mL of 1x Wash Buffer were carried out. The next step involved 21 µL of End Prep Reaction Buffer and 9 µL of End Prep Enzyme Mix combined with 150 µL of SPCs, and the distribution of the final volume into two PCR tubes. The reaction consisted of incubating the mix on a thermocycler set to 25ºC for 30 minutes, followed by 65ºC for 30 minutes. Once the DNA end was prepared, the content of the two tubes was pooled into the same tube and 3 washes with 1 mL of Wash Buffer were performed. The barcode ligation step was performed immediately after.

The Split-and-Pool barcoding protocol uses Atrandi's 96-well barcoding plate system, comprising four successive rounds of the same procedure on four different plate sections. The procedure consisted of preparing a master mix that included 150 µL of SPCs, 60 µL of Ligation Buffer, 20 µL of Ligation Enzyme, and 70 µL of NFW. This mixture was distributed in 24 wells of the plate-defined section and the incubation was performed at room temperature for 15 minutes. Finally, 40 µL of Stop Buffer were added to each well and incubated for 5 minutes to inhibit the reaction. The content of the wells treated was collected

and washed with 1 mL of 1x Wash Capsule in order to restart the process in the next section.

Once the ligation was carried out in the four defined sections, the pooled SPCs were dissolved to obtain the DNA barcoded content. The SPCs Generation Kit includes a Release Reagent that breaks the SPCs structures by adding 4 µL per each 100 µL of SPCs and incubating the mixture for 5 minutes at room temperature. Then, DNA was purified with the AMPure XP purification protocol, using 0.5x beads to sample volume ratio. This DNA sample was sent to the Sequencing Service of Fisabio for sequencing with the PacBio platform.
